# Oxidative and Proteolytic Inactivation of Alpha-1 Antitrypsin in Bronchopulmonary Dysplasia Pathogenesis: A Top-Down Proteomic Bronchoalveolar Lavage Fluid Analysis

**DOI:** 10.3389/fped.2021.597415

**Published:** 2021-03-23

**Authors:** Chiara Tirone, Federica Iavarone, Milena Tana, Alessandra Lio, Claudia Aurilia, Simonetta Costa, Massimo Castagnola, Irene Messana, Giovanni Vento

**Affiliations:** ^1^Dipartimento di Scienze della Vita e Sanità Pubblica, Unità Operativa Complessa di Neonatologia, Fondazione Policlinico Universitario A. Gemelli, Istituto di Ricovero e Cura a Carattere Scientifico, Rome, Italy; ^2^Dipartimento di Scienze Biotecnologiche di base, Cliniche Intensivologiche e Perioperatorie, Università Cattolica del Sacro Cuore, Rome, Italy; ^3^Fondazione Policlinico Universitario A. Gemelli, Istituto di Ricovero e Cura a Carattere Scientifico, Rome, Italy; ^4^Laboratorio di Proteomica e Metabonomica-Istituto di Ricovero e Cura a Carattere Scientifico Fondazione Santa Lucia, Rome, Italy; ^5^Istituto di Scienze e Tecnologie Chimiche “Giulio Natta,” Consiglio Nazionale delle Ricerche, Rome, Italy; ^6^Università Cattolica del Sacro Cuore, Istituto di Clinica Pediatrica, Rome, Italy

**Keywords:** alpha-1 antitrypsin, preterm infants, bronchopulmonary displasia, bronchoalveolar lavage fluid, proteomics

## Abstract

The study investigates the role of the oxidative and proteolytic inactivation of alpha-1 antitrypsin (AAT) in the pathogenesis of bronchopulmonary dysplasia (BPD) in premature infants. Bronchoalveolar lavage fluid (BALF) samples were collected on the 3rd day of life from mechanically ventilated neonates with gestational age ≤ 30 weeks and analyzed without previous treatment (top-down proteomics) by reverse-phase high-performance liquid chromatography-electrospray ionization mass spectrometry. AAT fragments were identified by high-resolution LTQ Orbitrap XL experiments and the relative abundances determined by considering the extracted ion current (XIC) peak area. Forty preterm neonates were studied: 20 (50%) did not develop BPD (no-BPD group), 17 (42.5%) developed mild or moderate new-BPD (mild + moderate BPD group), and 3 (7.5%) developed severe new-BPD (severe BPD group). Eighteen fragments of AAT and a fragment of AAT oxidized at a methionine residue were identified: significantly higher values of AAT fragments 25–57, 375–418, 397–418, 144–171, and 397–418 with oxidized methionine were found in the severe BPD group. The significantly higher levels of several AAT fragments and of the fragment 397–418, oxidized in BALF of preterm infants developing BPD, underlie the central role of an imbalance between proteases and protease inhibitors in exacerbating lung injury and inducing most severe forms of BPD. The study has some limitations, and between them, the small sample size implies the need for further confirmation by larger studies.

## Introduction

Bronchopulmonary dysplasia (BPD) is one of the most frequent chronic diseases of premature infants, causing ongoing respiratory morbidity and mortality (1). The pathogenesis of new BPD is multifactorial (2).

Our study group has been involved from several years in the identification of peptides present in the airways of preterm infants, with and without BPD, by using proteomic techniques (3).

An imbalance between proteases and protease inhibitors seems to contribute to the pathogenesis of new BPD (4).

Alpha-1 antitrypsin (AAT) is the best characterized inhibitor of neutrophil elastase (NE), a family of proteolytic enzymes implicated in the pathogenesis of BPD. A greater NE activity is consequent to AAT proteinase degradation and inactivation. The potential therapeutic use of AAT in numerous important diseases is due to its anti-inflammatory properties (5). Concerning BPD, an adequate AAT elastase inhibitory activity has been demonstrated in the airways of a new BPD baboon model but not in the severe BPD one (6). The same authors performed a treatment with a catalytic antioxidant in “severe BPD” baboon obtaining an increase of AAT elastase inhibitory activity. This result suggested that the pulmonary outcomes in animal models of “severe BPD” could be prevented by the oxidative inactivation of AAT due to antioxidant therapy.

Therefore, the objective of this study was to investigate the role of the oxidative and proteolytic inactivation of AAT, through the identification and determination of the relative abundance of AAT fragments and AAT fragments with methionine oxidation (Met-Ox) in bronchoalveolar lavage fluid (BALF) of premature infants with and without BPD. A top-down petidomic strategy aimed at identifying naturally occurring peptides and proteins of BALF represented the analytical approach. This method was conducted by reverse-phase high-performance liquid chromatography (RP-HPLC) coupled to electrospray ionization high-resolution mass spectrometry (ESI-HR-MS).

## Materials and Methods

### Human Participants

The study was carried out in the Neonatal Intensive Care Unit of the Fondazione Policlinico Universitario Agostino Gemelli in Rome and included 40 premature neonates with gestational age (GA) ≤30 weeks consecutively admitted between May 2013 and January 2016. They were eligible when (i) they were born in our hospital, (ii) endotracheal intubation was required at birth, and (iii) on-going intensive care and mechanical ventilation were required. Newborns with major congenital malformations, prenatal infection (positive blood and/or BALF culture at birth), or infants enrolled into the study but died before BPD diagnosis was made were all excluded from final analysis. Surfactant (a pig-derived natural surfactant, Curosurf, Chiesi Farmaceutici, Parma, Italy) was administered to all studied newborns, at a dose of 200 mg/kg, always in the neonatal unit. All the babies were ventilated in elective high-frequency oscillatory ventilation modality with Draeger Babylog 8000 plus (Draeger, Lubeck, Germany), as previously described (7), and received ibuprofen therapy if the ductus arteriosus was hemodynamically significant (8).

The study protocol and consent forms were approved by the Ethics Committee of the Department of Pediatrics, and the parents gave their informed consent.

The diagnosis of BPD was based on the need for supplementary oxygen for at least 28 days after birth, and its severity was determined according to the respiratory support required at 36 postmenstrual weeks: (1) mild BPD, breathing air; (2) moderate BPD, need for supplementary oxygen <30%; and (3) severe BPD, need for supplementary oxygen ≥30% and/or continuous positive airway pressure (CPAP) or ventilation (1). Patients enrolled have been divided into three groups according to the BPD diagnosis: no-BPD group, mild + moderate BPD group, and severe BPD group. Neonates with diagnosis of mild and moderate BPD have been considered together according to the results of a recent study showing that children with mild BPD exhibited at 6–8 years similar impairments in respiratory mechanics and lung structure to those diagnosed with moderate BPD, while children with initial diagnosis of severe BPD had the most impaired lung function test (9). The incidence of other clinical outcomes was also assessed: sepsis (defined as the presence of clinical signs of infection with a positive blood culture), severe intracranial hemorrhage (grade III or IV), necrotizing enterocolitis (stage > 2), ductus arteriosus surgically ligated, pneumonia (the diagnosis was based on the worsening of respiratory status—increase of fraction of inspired oxygen and/or ventilator setting—with increased amounts of secretions by endotracheal tube, rales, wheezing, persistent chest radiographic abnormalities, and a positive BALF culture) (10), duration of mechanical ventilation, O_2_-therapy, and survival to discharge.

### Sample Collection and Treatment

BALF samples were obtained on the 3rd day of life according to a standardized procedure (7). After collection, specimens were centrifuged at 1,000 g for 3 min and treated as previously described (11). Cells were suspended in 250 μl of 0.9% sodium chloride, and the absolute and differential cell counts were obtained by automatic analyzer (Bayer-ADVIA 120, Hematology System) and by spinning 25 μl of suspended cells onto two glass slides using May Grünwald-Giemsa stain, as previously described (11). Trifluoroacetic acid (TFA) 0.2% solution was immediately added to cell-free supernatants of BALF samples in 1:1 *v*/*v* ratio and solution centrifuged at 8,000 g for 5 min. The acidic treatment reduced the action of BALF proteases and artifact occurrence. After centrifugation, the acidic supernatants were separated from the precipitate, freeze-dried, and re-suspended in 300 μl of 0.2% trifluoroacetic acid solution; 100 μl of this soluble fraction from each sample were analyzed by HPLC-ESI-MS.

Moreover, BALF samples were cultured for microbiological analysis including bacteria, *Mycoplasma* spp., chlamydia, and fungi, in order to diagnose lung infection.

### Reagents and Instruments

All general chemicals and reagents were of analytical grade and were purchased from Merck (Damstadt, Germany) and J.T.Baker (Deventer, The Netherlands).

Low-resolution HPLC-ESI-MS measurements were carried out by a Surveyor HPLC system (Thermo Fisher, San Jose, CA, USA) connected by a T splitter to a photodiode-array detector and an LCQ Deca XP Plus mass spectrometer (Thermo Fisher). The mass spectrometer was equipped with an ESI source. High-resolution HPLC-ESI-MS/MS experiments were carried out by an Ultimate 3000 Micro HPLC apparatus (Dionex, Sunnyvale, CA, USA) equipped with a FLM-3000-Flow manager module and coupled to an LTQ Orbitrap XL apparatus (Thermo Fisher) (12).

### RP-HPLC-ESI-MS Separations and Qualitative Analysis

Low-resolution RP-HPLC-ESI-MS separations were performed by using a Zorbax SB300 C8 (Agilent) chromatographic column, with 5-μm particle diameter (column dimensions 150 × 2.1 mm). The following solutions were utilized for the low-resolution chromatographic separation: (eluent A) 0.056% aqueous TFA and (eluent B) 0.050% TFA in acetonitrile–water 80/20 (*v*/*v*). The applied gradient was linear from 0 to 55% of B in 40 min, at a flow rate of 0.30 ml/min. The T splitter permitted 0.20 ml/min to flow toward the diode array detector and 0.10 ml/min toward the ESI source. The photodiode array detector was set at a wavelength of 214 and 276 nm. During the first 5 min of separation, the eluate was not addressed to the mass spectrometer to avoid source contamination and instrument damage due to the high salt concentration. Mass spectra were collected every 3 ms in the positive ion mode. MS spray voltage was 4.50 kV and capillary temperature was 220°C (12).

High-resolution RP-HPLC-ESI-MS separations were performed by using a Zorbax 300 SB-C8 chromatographic column (150 mm × 1 mm) with 3.5-μm particle diameter and the following eluents: (eluent A) 0.01% formic acid in water and (eluent B) 0.01% formic acid in acetonitrile–water 80/20 (*v*/*v*). The applied gradient was as follows: 0–4 min 5% B, 4–34 min from 5 to 50% B (linear), and 34–64 min from 50 to 90% B (linear), at a flow rate of 80 μl/min. High-resolution positive MS/MS spectra were collected in data-dependent acquisition mode; the three most intense multiply-charged ions were selected and fragmented by collision-induced dissociation (35% normalized collision energy) and spectra were recorded. Tuning parameters were as follows: capillary temperature 250°C, source voltage 5 kV, capillary voltage 23 V, and tube lens voltage 35 V.

HPLC-ESI-MS LTQ Orbitrap XL data were elaborated by the Proteome Discoverer 1.2 program, based on SEQUEST cluster as search engine (University of Washington, USA, licensed to Thermo Electron Corp., San Jose, CA, USA) against Swiss-Prot human proteome (November 11th, 2011 released; uniprot-taxonomy-9606-AND-reviewed-yes.fasta; 73,521 non-redundant protein sequences). Experimental mass values were compared with theoretical mass values available at the Swiss-Prot data bank (http://us.expasy.org/tools).

### Quantification of AAT Fragments

**Figure 1:**
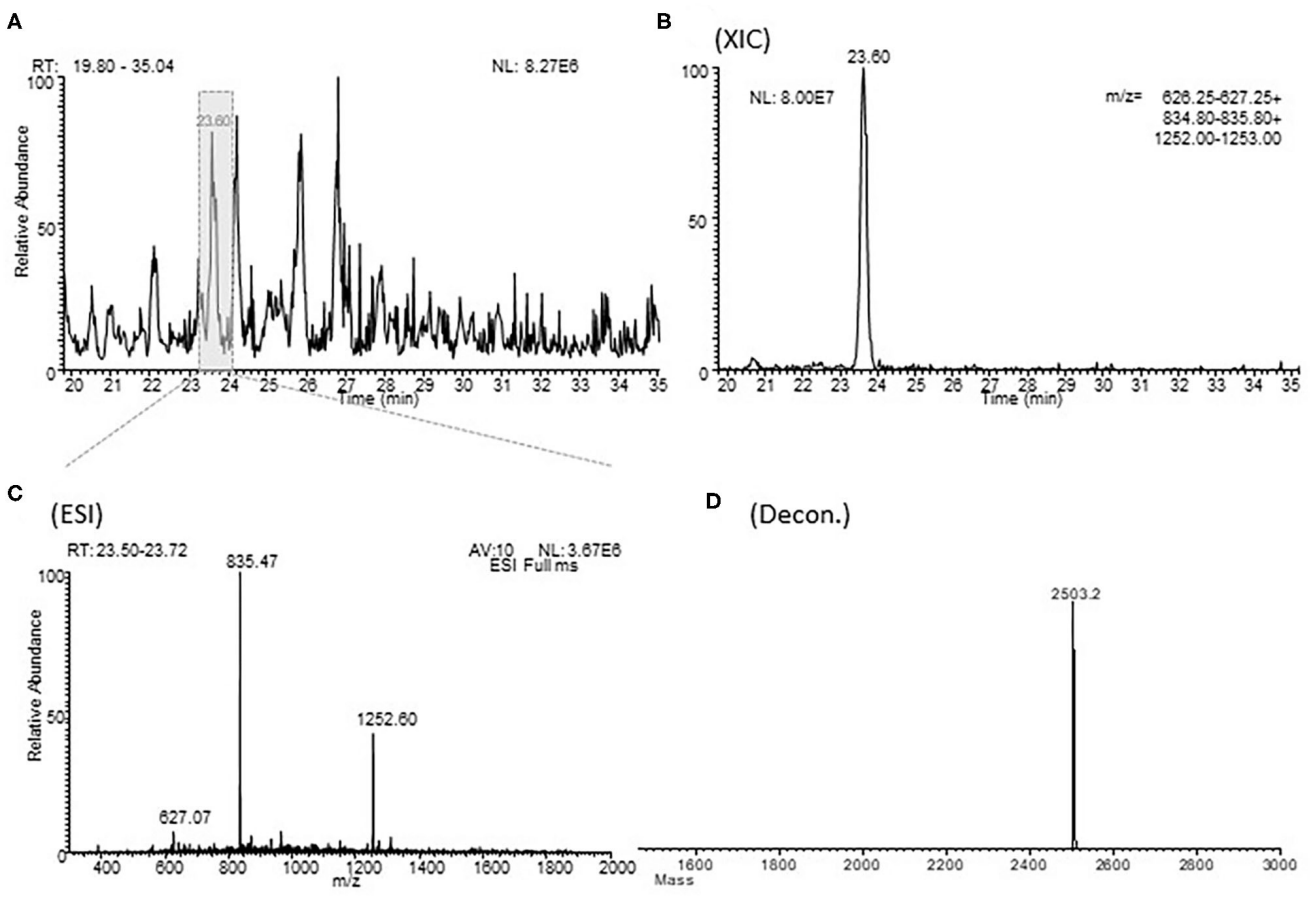
Example of a XIC procedure for the relative quantitation of the fragment 397-418 of alpha-1 antitrypsin in one typical sample of BALF. **(A)** shows an enlargement of the total ion current chromatographic profile of a sample of BALF in the range of 20.0-35.0 min. **(B)** (XIC) shows the extraction from the HPLC-ESI-MS profile of the *m/z* values characteristic of fragment 397-418 of alpha-1 antitrypsin. **(C)** (ESI) shows the corresponding ESI spectra and the **(D)** (Decon) shows his deconvolution. RT, retention time; MA, measured area; AV, average; NL, normalization level.

The relative abundances of the different AAT fragments were determined by measuring the extracted ion current (XIC) peak area that, under identical experimental conditions, is linearly proportional to peptide concentration and can be used with confidence to monitor relative abundances (13). The XIC procedure is based on the extraction of the current associated to either one or multiple selected ions from the total ion current (TIC) chromatographic profile. In the determination of XIC peak area, a correct choice of the *m*/*z* values for protein detection is necessary, avoiding *m*/*z* potentially overlapping with other close-eluting proteins (14) (Figure 1).

According to the ERS Task Force recommendations for bronchoalveolar lavage in children (15), we reported the data as volume instilled, volume recovered, and concentration per mL.

### Statistical Analysis

Categorical variables were compared by using a chi-square test. Testing for differences of continuous variables between the three groups was accomplished by one-way analysis of variance (ANOVA) test. The statistical software used included Instat (GraphPad PRISM Version 5). A *p* < 0.05 was considered statistically significant.

## Results

During the study period, 40 neonates were enrolled: 20 (50%) did not develop BPD (no-BPD group), 17 (42.5%) developed a mild (*N* = 13) or moderate (*N* = 4) new-BPD (mild + moderate BPD group), and 3 (7.5%) developed a severe new-BPD (severe BPD group).

A single BALF sample was studied for each neonate, and the range of BALF recovery was 75–80% of the instilled volume (2 ml/kg of 0.9% sodium chloride) in all the babies. The characteristics of patients and their major outcomes are summarized in Tables 1, 2.

**Table 1:** Patient characteristics.

	**No BPD group (*N* = 20)**	**Mild + moderate BPD group (*N* = 17)**	**Severe BPD group (*N* = 3)**
Gestational age (weeks)^**^	27.8 ± 1.4	25.9 ± 1.3	26.7 ± 2.5
Birth weight (grams)^**^	973 ± 226	772 ± 184	838 ± 357
Male^**^	6 (30)	12 (70)	2 (67)
Small for gestational age	3 (14)	5 (29)	1 (33)
Antenatal steroids^*^	11 (55)	8 (47)	0 (0)
Vaginal delivery	1 (5)	4 (23)	1 (33)
Rupture of membranes ≥ 12 h^**^	4 (20)	4 (23)	3 (100)
Apgar score 1′	6 (1–8)	4 (2–8)	3 (3–5)
Apgar score 5′	8 (4–9)	7 (5–9)	7 (7–7)

*Values expressed as mean ± SD and no. (%)*.

* *We included only the newborns receiving a completed course of prenatal betamethasone, defined as two doses administered more than 24 h, but no more than 7 days before delivery*.

** *p < 0.05*.

**Table 2:** Clinical outcomes in the studied newborns.

	**No BPD group (*N* = 20)**	**Mild + moderate BPD group (*N* = 17)**	**Severe BPD group (*N* = 3)**
Sepsis	4 (20)	10 (59)	2 (67)
Necrotizing enterocolitis stage >2	3 (14)	1 (6)	1 (33)
Pneumonia^**^	7 (35)	14 (82)	3 (100)
Ductus arteriosus surgically ligated^**^	0 (0)	9 (53)	1 (33)
Intracranial hemorrhage grade III or IV	1 (5)	4 (23)	1 (33)
Duration of mechanical ventilation (hours)^**^	103 ± 76	582 ± 206	786 ± 683
Duration of O_2_-therapy (hours)^**^	174 ± 209	1,300 ± 529	3,508 ± 726
Survival to discharge	20 (100)	16 (94)	3 (100)

*Values expressed as mean ± SD, no. (%)*.

** *p ≤ 0.05*.

The no-BPD group had a significantly higher GA compared to the other two groups (*p* < 0.05). The number of male neonates was significantly lower in the no-BPD group with respect to the others, and the incidence of rupture of membranes was significantly higher in the severe BPD group (*p* < 0.05, Table 1). The incidence of sepsis and intracranial hemorrhage grade III or IV was less represented in the no-BPD group with respect to the mild + moderate BPD and severe BPD groups, but the difference was not statistically significant. The no-BPD group had a significantly lower incidence of pneumonia and of persistence of ductus arteriosus (PDA) requiring surgical ligation, with respect to the other two groups. The duration of mechanical ventilation and O_2_ therapy was significantly longer in the severe BPD group if compared to the other two groups (Table 2).

The median (range) absolute leukocyte count resulted to be higher in the severe BPD group [2.78 (1.25–4.87) leukocytes × 10^9^/l) with respect to the mild + moderate BPD [1.92 (0.59–4.5) leukocytes × 10^9^/l) and no-BPD groups [1.83 (0.35–11.00) leukocytes × 10^9^/l), even if the difference was not statistically significant (*p* = 0.79). The highest value of absolute neutrophil count was found in the severe BPD group [2.58 (1.06–4.14) neutrophils × 10^9^/l] with respect to the other two groups [1.55 (0.24–7.70) neutrophils × 10^9^/l and 1.34 (0.50–3.80) neutrophils × 10^9^/l in the no-BPD group and in the mild + moderate BPD group, respectively], but the difference was not statistically significant (*p* = 0.51).

The RP-HPLC-ESI-MS analysis of the BALF samples showed the presence of 18 peptides attributable to AAT fragments. Their average mass and levels (XIC peak areas) are shown in Table 3. The methionine sulfoxide (Met-Ox) derivative (2,519.3 Da) of the fragment 397–418 was also characterized. Significantly higher levels of the AAT fragments 25–57, 375–418, 397–418, 397–418 (Met-Ox), and 144–171 were found in the severe BPD group with respect to the mild + moderate BPD and the no-BPD groups (Table 3). The mean ± SD FiO_2_ in the first 3 days of life (before BALF sample collection) was higher in the severe BPD group with respect to the no-BPD and the mild + moderate BPD groups, with a statistically significant difference in the 2nd day of life (mean ± SD: 0.26 ± 0.09 in the no-BPD group; 0.25 ± 0.07 in the mild + moderate BPD group, and 0.42 ± 0.20 in the severe BPD group; *p* = 0.02).

**Table 3:** AAT fragments identified in BALF samples and mean XIC peak area value (±SD) determined in the three groups under study.

**Fragments of AAT (Mav)**	**No BPD group (*N* = 20) (AU × 10^**8**^)**	**Mild + moderate BPD group (*N* = 17) (AU × 10^**8**^)**	**Severe BPD group (*N* = 3) (AU × 10^**8**^)**
Fragment 25–57 (3,704.7)^**^	1.1 ± 1.8	4.2 ± 6.5	29.9 ± 30.0
Fragment 375–418 (5,068.1)^**^	0.4 ± 0.8	1.4 ± 1.8	4.8 ± 3.5
Fragment 397–418 Met-Ox (2,519.3)^**^	0.8 ± 1.6	1.5 ± 2.6	15.3 ± 16.5
Fragment 397–418 (2,503.3)^**^	1.6 ± 2.2	2.9 ± 4.0	7.9 ± 9.1
Fragment 144–171 (3,269.8)^**^	0.2 ± 0.6	0.6 ± 0.3	3.3 ± 4.2
Fragment 383–396 (1,648.9)	0.1 ± 0.3	1.2 ± 0.2	0.2 ± 0.3
Fragment 109–123 (1,740.8)	1.4 ± 0.4	0.4 ± 1.4	0 ± 0
Fragment 172–184 (1,510.7)	0.6 ± 1.3	1.9 ± 3.7	0.9 ± 0.9
Fragment 212–226 (1,926.0)	1.0 ± 0.1	0.2 ± 0.6	0 ± 0
Fragment 377–396 (2,303.3)	0.4 ± 0.8	1.0 ± 2.4	1.4 ± 1.8
Fragment 354–376 (2,374.2)	0.4 ± 1.7	0.3 ± 1.0	0.1 ± 0.2
Fragment 185–206 (2,489.4)	1.1 ± 2.3	0.6 ± 1.2	2.0 ± 2.0
Fragment 30–54 (2,831.3)	0.02 ± 0.07	0.1 ± 0.3	0 ± 0
Fragment 343–376 (3,498.9)	0.03 ± 0.14	0.09 ± 0.26	0 ± 0
Fragment 316–353 (3,922.1)	0.3 ± 0.6	0.4 ± 0.7	0.8 ± 1.4
Fragment 383–418 (4,133.2)	0.3 ± 0.6	0.7 ± 1.5	0.2 ± 0.3
Fragment 185–226 (4,956.7)	0.1 ± 0.3	0.6 ± 1.3	0 ± 0
Fragment 316–376 (6,277.3)	0.1 ± 0.4	0.6 ± 1.7	0.6 ± 0.7
Fragment 124–171 (5,466.9)	0 ± 0	0.3 ± 1.2	0 ± 0

*AU, arbitrary unit; Mav, average mass in Dalton*.

** *p ≤ 0.01*.

## Discussion

To date, no molecules able to definitively predict the predisposition to develop BPD before its clinical onset have been characterized. Many studies conducted in the past years have shown that an imbalance between protease and antiprotease activity is central to the pathogenesis of BPD. The activity of serine protease inhibitor B1 (SERPINB1), which belongs to a subgroup of serpins with unique inhibitory specificity for both NE and cathepsin G, has been evaluated in the “old/severe BPD” and the “new BPD” models of preterm baboon (6, 16). Increased lung expression of SERPINB1 in the full-term baboons and in the new BPD model with lower SERPINB1 expression and higher NE activity levels in the old BPD model were found (16).

These results suggest that SERPINB1 upregulation in the new BPD plays an important role by participating in the regulation of neutrophil proteases (NE and cathepsin G) along with other well-known elastase inhibitors, such as AAT and secretory leukocyte protease inhibitor. The authors underlined the balance between NE and its inhibitors is favorably influenced by the surfactant treatment and the subsequent decreased use of supplemental oxygen. In fact, as a consequence, the oxidative inactivation of NE inhibitors, such as AAT and SERPINB1, is limited.

Karaaslan et al. (6) investigated the elastase inhibitory activity of AAT in the same two baboon models of BPD and determined the effect of treatment with a catalytic antioxidant. A sufficient elastase inhibitory activity of the airway AAT was demonstrated in the new BPD model, but not in the severe one. An increase of the elastase inhibitory activity of AAT was obtained by treating with the catalytic antioxidant the severe BPD group baboons. Therefore, in animal models of severe BPD, the prevention of oxidative inactivation of AAT can represent one of the mechanisms by which antioxidant therapy improves the pulmonary outcomes.

The intravenous administration of AAT has been tested in neonates with respiratory distress syndrome treated with surfactant (17). However, in this study, a reduction in the incidence of pulmonary hemorrhage rather than BPD was obtained. Probably, these results depend on the low levels of NE in the lungs of the “new BPD” neonates.

In the present study, significantly higher levels of the fragments 25–57, 375–418, 397–418, 397–418 (Met-Ox), and 144–171 of AAT were found in the severe BPD group with respect to the mild + moderate BPD group and the no-BPD group, most likely dependent on a major AAT proteolytic degradation or AAT oxidative inactivation.

The inactivation of AAT by oxidation of either methionine 351 or 358 demonstrated to be a mechanism for activity regulation at sites of inflammation (18). According to the higher levels of the oxidized fragment 397–418 in our severe BPD group patients, their mean FiO_2_ value was higher in each of the first 3 days of life, with respect to the other two groups, even if the difference was statistically significant only in the 2nd day of life. It is most likely that the higher levels of the oxidized AAT fragment in the neonates, who will develop a severe new BPD form, represent the effect of a cumulative free radical action due to the exposition to higher FiO_2_ in the 1st days of life.

We performed a search on MEROPS peptidase database (http://merops.sanger.ac.uk/) to determine which might potentially be the major proteases responsible for AAT degradation. We obtained some interesting results regarding the cleavages at position 57–58 (Glu-Phe + Ala), potentially operated by pepsin, and 396–397 (Val-Phe + Leu), potentially operated by matrix metallopeptidase-7.

Pepsin is a gastric proteolytic enzyme, already found increased by Farhath et al. (19) in the tracheal aspirates of preterm infant who developed severe BPD, secondary to gastric aspiration. Matrix metallopeptidase-7, described as responsible for generating unique proteolitic fragment of alpha 1-antitrypsin in ductal fluid of breast cancer patients (20), could be the peptidase involved in the formation of the fragment 397–418 in our patients.

The fragment 375–418 could be generated by matrix metallopeptidase-11, which allows the release of a bioactive peptide as previous largely described (21). No potentially major proteases responsible for the generation of the AAT fragment 144–171 has been found and could be the object of future studies.

Concerning the absolute number of neutrophils in the BALF, no significant differences were found among the three groups, even if the highest absolute neutrophil count value was found in the severe BPD group, along with the production of larger amounts of AAT fragments. These findings should testify that neutrophilic activation has occurred with the production of NE. As described in our previous work (22), we could speculate that neutrophilic activation with consequent NE production occurred earlier than neutrophil recruitment. In fact, most of our patients (30/40 = 75%) were extubated during the 1st week of life, and BALF samples were not collected after the 3rd day.

This study has some limitations: (i) the small number of the studied patients and particularly of the severe BPD ones, even if the differences were statistically significant, and (ii) the volume of BALF samples was insufficient to evaluate the total AAT activity. This does not allow establishing if the higher concentration of AAT fragments depends on an enhanced diffusion of the AAT molecule from the serum. Anyway, a highest absolute neutrophil count in the severe BPD group has emerged even if the difference is not statistically significant. This finding could, at least in part, attest that the higher levels of AAT fragments really depend on the neutrophil production.

In conclusion, the results of the present study confirm *in vivo* what has been experimentally found in baboon models of old and new BPD (6, 16), by demonstrating a major AAT proteolytic and/or oxidative inactivation in the neonates who develop a severe new-BPD form.

In the future, it could be useful to carry out studies aimed at experimentally verifying the implications of AAT degradation in the pathogenesis of BPD. If this is confirmed, it may be of interest to verify the efficacy of increasing AAT antiprotease activity by aerosol or intravenous administration or by treatment with a catalytic antioxidant in infants who develop severe BPD.

## Data Availability Statement

The raw data supporting the conclusions of this article will be made available by the authors, without undue reservation.

## Ethics Statement

The studies involving human participants were reviewed and approved by Comitato etico, Fondazione Policlinico Universitario A. Gemelli, IRCCS, Rome, Italy. Written informed consent to participate in this study was provided by the participants' legal guardian/next of kin.

## Author Contributions

CT designed the study, collected samples, analyzed the data, and wrote the first and final drafts of the manuscript. FI designed the study, performed the proteomic analysis, analyzed the data and wrote the first and final drafts of the manuscript. MT, AL, CA, and SC carried out the data collection and the literature search, assisted with the analysis, and provided significant edits to the manuscript. IM, MC, and GV conceptualized and designed the study and critically reviewed the manuscript. All authors read and approved the submission of this version of the manuscript.

## Conflict of Interest

The authors declare that the research was conducted in the absence of any commercial or financial relationships that could be construed as a potential conflict of interest.

## References

[1] Jobe AH Bancalari E. Bronchopulmonary Dysplasia. Am J Respir Crit Care Med. (2001) 163:1723–9. 10.1164/ajrccm.163.7.2011060 11401896

[2] Jobe AH. The new bronchopulmonary dysplasia. Curr Opin Pediatr. (2011) 23:167–72. 10.1097/MOP.0b013e3283423e6b 21169836 PMC3265791

[3] Vento G Tirone C Lulli P Capoluongo E Ameglio F Lozzi S. Bronchoalveolar lavage fluid peptidomics suggests a possible matrix metalloproteinase-3 role in bronchopulmonary dysplasia. Intensive Care Med. (2009) 35:2115–24. 10.1007/s00134-009-1646-6 19779697

[4] Davies PL Spiller OB Beeton ML Maxwell NC Remold-O'Donnell E Kotecha S. Relantionship of proteinases and proteinase inhibitors with antimicrobial presence in chronic lung disease of prematurity. Thorax. (2010) 65:246–51. 10.1136/thx.2009.116061 20335295 PMC2921268

[5] Bergin DA Hurley K McElvaney N Reeves EP. Alpha-1 Antitrypsin: a potent anti-inflammatory and potential novel therapeutic agent. Arch Immunol Ther Exp (Warsz). (2012) 60:81–97. 10.1007/s00005-012-0162-5 22349104

[6] Karaaslan C Hirakawa H Yasumatsu R Chang LL Pierce RA Crapo JD. Elastase inhibitory activity of airway α1- antitrypsin is protected by treatment with a catalytic antioxidant in a baboon model of severe bronchopulmonary dysplasia. Pediatr Res. (2011) 70:363–7. 10.1203/PDR.0b013e31822a357e 21705962 PMC3166355

[7] Vento G Matassa PG Ameglio F Capoluongo E Zecca E Tortorolo L. HFOV in premature neonates: effects on pulmonary mechanics and epithelial lining fluid cytokines. A randomized controlled trial. Intensive Care Med. (2005) 31:463–70. 10.1007/s00134-005-2556-x 15717206

[8] Su BH Watanabe T Shimizu M Yanagisawa M. Echocardiographic assessment of patent ductus arteriosus shunt flow pattern in premature infants. Arch Dis Child Fetal Neonatal Ed. (1997) 77:F36–40. 10.1136/fn.77.1.F36 9279181 PMC1720677

[9] Broström EB Thunqvist P Adenfelt G Borling E Katz-Salamon M. Obstructive lung disease in children with mild to severe BPD. Respir Med. (2010) 104:362–70. 10.1016/j.rmed.2009.10.008 19906521

[10] National Nosocomial Infection Survey Manual. Available online at: http://www.cdc.gov/ncidod/dhqp/pdf/nhsn/NHSNManualPatientSafetyProtocol052407.pdf (accessed November 16, 2009).

[11] Vento G Matassa PG Zecca E Tortorolo L Martelli M De Carolis MP. Effect of dexamethasone on tracheobronchial aspirate fluid cytology and pulmonary mechanics in preterm infants. Pharmacology. (2004) 71:113–9. 10.1159/000077444 15161992

[12] Castagnola M Inzitari R Fanali C Iavarone F Vitali A Desiderio C. The surprising composition of the salivary proteome of preterm human newborn. Mol Cell Proteomics. (2011) 10:M110.003467. 10.1074/mcp.M110.003467 20943598 PMC3013458

[13] Levin Y Schwarz E Wang L Leweke FM Bahn S. Label-free LC-MS/MS quantitative proteomics for large-scale biomarker discovery in complex samples. J Sep Sci. (2007) 30:2198–203. 10.1002/jssc.200700189 17668910

[14] Ong SE Mann M. Mass spectrometry-based proteomics turns quantitative. Nat Chem Biol. (2005) 1:252–62. 10.1038/nchembio736 16408053

[15] de Blic J Midulla F Barbato A Clement A Dab I Eber E. Bronchoalveolar lavage in children. ERS Task Force on bronchoalveolar lavage in children. European Respiratory Society. Eur Respir J. (2000) 15:217–31. 10.1183/09031936.00.15121700 10678650

[16] Yasumatsu R Altiok O Benarafa C Yasumatsu C Bingol-Karakoc G Remold-O'Donnell E. SERPINB1 upregolation is associated with *in vivo* complex formation with neutrophil elastase and cathepsin G in baboon model of bronchopulmonary dysplasia. Am J Physiol Lung Cell Mol Physiol. (2006) 291:L619–27. 10.1152/ajplung.00507.2005 16617093

[17] Stiskal JA Dunn MS Shennan AT O'Brien KK Kelly EN Koppel RI. α1-Proteinase Inhibitor therapy for the prevention of chronic lung disease of prematurity: a randomized controlled trial. Pediatrics. 101(1 Pt 1):89–94. 10.1542/peds.101.1.89 9417158

[18] Taggart C Cervantes-Laurean D Kim G McElvaney NG Wehr N Moss J. Oxidation of either Methionine 351 or Methionine 358 in α1-antitrypsin causes loss of anti-neutrophil elastase activity. J Biol Chem. (2000) 1:275:27258–65. 10.1016/S0021-9258(19)61505-X 10867014

[19] Farhath S He Z Nakhla T Saslow J Soundar S Camacho J. Pepsin, a marker of gastric contents, is increased in tracheal aspirates from preterm infants who develop bronchopulmonary dysplasia. Pediatrics. (2008) 121:e253–9. 10.1542/peds.2007-0056 18245400

[20] Zhou J Trock B Tsangaris TN Friedman NB Shapiro D Brotzman M. A unique proteolytic fragment of alpha1-antitrypsin is elevated in ductal fluid of breast cancer patient. Breast Cancer Res Treat. (2010) 23:73–86. 10.1007/s10549-009-0625-5 19902353

[21] Niemann MA Baggott JE Miller EJ. Binding of SPAAT, the 44-residue C-terminal peptide of alpha 1-antitrypsin, to proteins of the extracellular matrix. J Cell Biochem. (1997) 1:66:346–57. 10.1002/(SICI)1097-4644(19970901)66:3<346::AID-JCB7>3.0.CO 9257191

[22] Vento G Lio A Tirone C Aurilia C Tana M Piras A. Association of high levels of α-defensins and S100A proteins with Candida mannan detection in bronchoalveolar lavage fluid of preterm neonates. Pediatr Res. (2013) 74:19–25. 10.1038/pr.2013.60 23575874

